# Stigmatization of Overweight and Obese Peers among Children

**DOI:** 10.3389/fpsyg.2017.00524

**Published:** 2017-04-20

**Authors:** Roberta Di Pasquale, Laura Celsi

**Affiliations:** Department of Human Sciences, Università degli Studi di BergamoBergamo, Italy

**Keywords:** children, overweight, obesity, stigma, peer discrimination

## Abstract

Children get involved in social categorization. Thus, they are able to stigmatize peers as well as to show in-group favoritism theorized by Tajfel and Turner (1986). Moreover, according to Aboud's Cognitive-Developmental Theory (1988, 2003) the intensity of children's stereotypes and negative attitudes toward socially devalued group members changes with age, in line with their cognitive development. In our Western society, which addresses especially females with the message that thinness is beauty, self-efficacy, power, and success, being overweight or obese is one of the most socially devalued and stigmatized conditions among children. Thus, overweight and obese children are more likely to be personally and socially devalued compared to their average size peers. Starting with these theoretical reflections, the objectives of this mini-review are to examine if: (1) obese children show in-group favoritism and thus show less anti-fat attitudes than their thin and normal weight peers; (2) fat stigma is more prevalent toward overweight and obese girls than toward boys; (3) the intensity of weight-related stigma changes with the cognitive development of children.

## Introduction

According to Tajfel and Turner (1986), social categorization is a process that allows people to analyze the environment, act efficiently within it, and to identify people's position in society, in relation to the value socially attributed to their group membership. The outcome of this process is the social identity, the part of one's self-concept that derives from group memberships. This self-concept is relational, relative, and comparative. Therefore, people's self-esteem depends on the positive or negative worth conferred to their group membership. Individuals who belong to devalued groups undergo stigmatization. Stigma is a set of negative beliefs and attitudes that are shown by bias, stereotypes, prejudices, rejection, and discrimination toward target groups. Independently from the value ascribed to a group, people tend to evaluate their in-group members more positively than out-group components.

Yee and Brown (1992) stated not only that children get involved in the social categorization process but also that when as young as 3 years old they show in-group favoritism.

Moreover, Aboud (1988, 2003) suggested that children's stereotypes change with their cognitive development. Specifically, per his Cognitive-Developmental Theory, young children's evaluations are dominated by the fear of the unfamiliar and are therefore crude and encompassing toward differences. By approximately 5–8 years of age, children's prejudice reach a peak, as they prefer their in-group to out-groups and then, from about 8 to 9 years of age, it decreases because children increasingly appreciate other people's perspectives.

Stigma, like categorization is context-dependent. Therefore, in different cultures and at different times, stigmatization may affect different groups. Today, in our Western society, obesity appears as one of the most stigmatizing and least socially acceptable conditions among children (Schwimmer et al., 2003). Several studies have highlighted that overweight and obese children are more likely to be victims of aggression than normal size peers and are frequently exposed to peers' intentional negative actions that are physical (e.g., kicking, pushing, hitting), verbal (e.g., being teased, name calling, derogatory remarks,) or relational (e.g., being ignored or avoided, social exclusion, being targets of rumors; Janssen et al., 2004; Griffiths et al., 2006; Puhl and Latner, 2007).

This phenomenon can be explained through the impact of the socially dominant esthetic ideals that equate thinness with beauty, self-efficacy, power, and success and that is pervasive especially for girls more than for boys (Grossi and Ruspini, 2007; Riva, 2012).

Based on the stimulus provided by the aforementioned theories the present review examines if:

1. Obese children show in-group favoritism and thus show less anti-fat attitudes than their thin and normal weight peers.
2. Fat stigma is more prevalent toward overweight and obese girls than toward boys.
3. The intensity of weight-related stigma changes as does the cognitive development of children.

## Methods

Electronic databases (PsycARTICLES, Scopus and Web of Science) were searched using the following 8 terms “victimization,” “anti-fat bias,” “anti-fat attitude,” “stigmatization,” “stigma,” “discrimination,” “prejudice,” “stereotype” combined with “obese children” or “obesity AND children.” Databases were investigated limiting the search to psychology and social work but excluding any restrictions about articles' year of publication.

Additional manuscripts were derived from the bibliography's analysis of eligible articles.

Studies were included in the review if: (1) the age of participants didn't exceed 11 years old^1^; (2) the weight-based stigmatization phenomenon was analyzed by the perpetrator's point of view and not by the victims' or by the observers' ones; (3) the main objective of the study was to assess the presence and the nature of this phenomenon instead of focusing on its causes and its effects.

## Results

**Figure 1:**
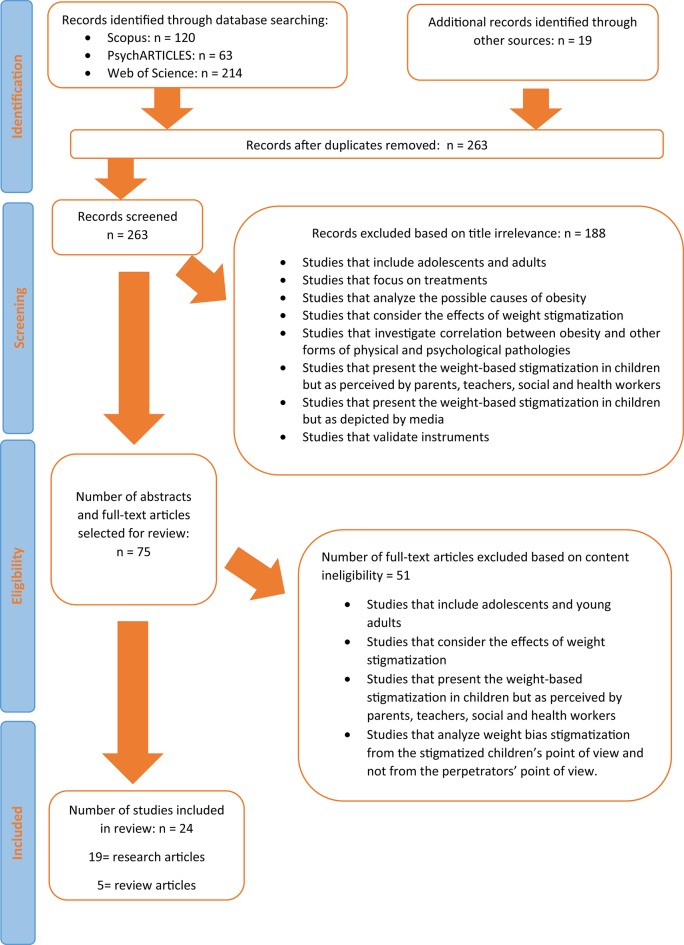
**Flow chart**.

As described in Figure 1, the initial search identified 416 papers. One hundred and fifty-three articles were removed due to duplication, 188 excluded upon review of title and another 38 left out after reading abstracts and full texts. The process resulted in 24 papers for inclusion in the review. Five articles were reviews and thus were not addressed in the results section.

### Overall study characteristics

As it can be inferred from Table 1, more than half of the studies took place in Europe (*n* = 11), 7 in the USA and 1 in New Zealand.

**Table 1:** **Overall study characteristics**.

**Authors**	**Country**	**Aim of the study**	**Sample (size and age range in years or mean age and were indicated, BMI)**	**Study design**	**Measures**	**Main findings**	**Limitations**
Lerner and Gellert, 1969	USA	To investigate: • Children's ability to recognize their body size • Children's preference for different body sizes.	45 children (24 males and 21 females) between 5.3 and 6.2 years old. Regarding ethnicity, all participants were white.	Cross-sectional	A body build preference task: children were shown three pictures of a thin, an average and a fat peer. Then they were asked to select the photograph that best depicts themselves, the one they would most want to look like and the one they would least like to look like. Finally, they were also asked to indicate same-sexed classmates who most resemble each picture.	Although no particular body build preference was shown by the majority, a consistent aversion to chubbiness was expressed by 86%. No gender differences were noted.	• The sample size • The lack of participants' BMI • The use of silhouettes that didn't correspond to specific BMIs
Counts et al., 1986	USA	To assess: • The role of facial attractiveness and body build on social perception among obese and normal weight children.	24 children (12 males and 12 females) between 8 and 11 years old. More in detail: • 6 third graders (50% boys) • 14 fourth graders (50%boys) • 4 fifth graders (50% boys) Regarding body build, 12 participants were obese and 12 weren't obese. Concerning ethnicity, all children were white, Caucasian.	Cross-sectional	• Subjective evaluation of body size • 2 adjective and behavior attribution tasks: first children were asked to evaluate an overweight 14-year-old student teacher regarding her attractiveness and her expected competence as a teacher. Then participants were show a photograph representing an average and a fat space person and were asked to attribute to them a set of 8 positive and negative qualities (better partner, smarter, friendlier, better leader, works better in a group, more sad, fights, works alone).	In the absence of facial attractiveness information, both normal and obese children selected the average space target as a better partner and leader than the overweight and obese ones. In the presence of facial attractiveness information, both normal and obese children viewed the obese target fairly positively. Actual body size didn't influence the selection task. No sex differences were revealed.	• The sample size • The lack of participants' BMI • The use of silhouettes that didn't perfectly correspond to specific BMIs • The lack of children's perceived body size
Goldfield and Chrisler, 1995	USA	To explore: • Children's stigmatization of fat peers	29 children (14 females and 15 males) 6 years of age. Concerning ethnicity participants were 12 Hispanic, 10 Caucasian, 6 African American and 1 Asian American.	Cross-sectional	• Body size perception: children were shown a set of body silhouettes and then they were asked to indicate which child they resemble most. • A friend selection task: children were shown the same set of body silhouettes and then they were asked which they would like most as a friend. • A body preference task: children were shown the same set of body silhouettes and then they were asked to indicate which child was the good one.	Many children (*f* = 28) were significantly less likely to say that the endomorphic child looked like them and significantly less likely to say they would want to be friends with the endomorphic child. No gender differences were noted.	• The number of participants • The lack of participants' BMI • The use of silhouettes that didn't perfectly correspond to a specific BMI
Cramer and Steinwert, 1998	USA	To investigate: • Anti-fat attitudes in children as young as 3–5 years old • Relationship between anti-fat attitudes and body size	113 preschool children between 3 and 5 years old.	Cross-sectional	4 measures to assess the obesity bias: • A simple adjective attribution task. • An adjective attribution task based on a story. • A personal body attitude task. • A playmate preference task.	Fat stigmatism was stronger in the older children, but it was clearly present in the 3 year olds. The cultural stereotype that 'fat is bad' was pervasive across gender, regardless of the child's own body build. In fact, overweight preschoolers demonstrated stronger stigmatism than did those who were not overweight.	• The lack of children's BMIs • The lack of children's perceived body size • The forced-choice paradigm
Turnbull et al., 2000	UK	To investigate: • Gender differences in anti-fat attitudes in preschool children	25 children (12 boys and 13 girls) aged between 2 and 5.	Cross-sectional	A force choice adjective attribution task to assess anti-fat bias realized through the use of dolls.	Children ascribed more negative characteristics than positive ones to fat figures than to normal figures, and more to fat female than to fat male figures.	• The sample size • The lack of participants' BMIs • The forced-choice paradigm • Considering participants as a large group instead of dividing them into different categories
Kraig and Keel, 2001	UK	To investigate: • If girls are subject to more negative anti-fat stigmatization than boys	34 children (19 girls and 15 girls) between 7 and 9 years old. Regarding BMI, subjects' mean BMI was 18.6 (3.5) kg/m^2^. Two children (6%) had BMIs that fell below the 5th percentile for pre-adolescent children, and one child (3%) had a BMI that fell above the 95th percentile for pre-adolescent children. Concerning ethnicity, 22 children (65%) were Caucasian, 10 (29%) had mixed ethnic backgrounds and 2 (6%) were Asian. Participants' socio-economic background was quite high.	Cross-sectional	• BMI • An adjective attribution task based on the methodology used by Staffieri. The unique differences were that in this study: 1) the illustrations to rate were 6:3 representing a thin, an average and an obese boy and 3 representing a thin, an average and an obese girl; 2) there were 9 descriptors. “Fat” was the one introduced.	Evaluations were most favorable for illustrations of thin children and least favorable for illustrations of chubby children. Children's BMI did not influence the pattern of their evaluations. Although girls didn't appear to be the object of a greater degree of social stigmatization compared to boys, this study revealed that when rating boys, children distinguished between being overweight vs. being normal weight or thin. When rating girls, however, children distinguished between being thin vs. being normal weight and overweight.	• The sample size • The limited range of socio-economic backgrounds • The disproportion between normal weight vs. average and thin children • The use of figures that don't correspond to specific BMI
Penny and Haddock, 2007a	UK	To investigate: • the mere proximity effect as a possible manifestation of anti-fat attitudes	Eighty-nine children (47 females and 42 males) aged between 5 and 10 years.	Cross-sectional	• A friend selection task realized through the use of wenty-four pairs of images specifically created for this study.	Anti-fat prejudice, with children preferring average-weight to overweight targets was evident. Second, overweight female targets were liked less than average-weight female targets, with no effect of target size for male targets. This is likely due to a higher emphasis being placed on female thinness. Third, target size and background size interacted to influence liking judgments of a female target, with an average-weight female target evaluated more negatively on an overweight background and an overweight female target evaluated more negatively on an average-weight background.	• The sample size • The lack of BMIs • The lack of children's perceived body size • The sample size The use of figures that don't correspond to specific BMI
Penny and Haddock, 2007b	UK	To investigate: • The presence of anti-fat attitudes in children • The predictions of the Cognitive-Developmental Theory (Aboud, 1988, 2003)	73 children (47 females and 26 males) between 5 and 10 years old, divided into three age groups: • 27 participants (18 females and 9 males) aged between 5 and 6 years; • 26 participants (16 females and 6 males) aged between 7 and 8 years; • 20 participants (13 females and 7 males) aged between 9 and 10 years.	Cross-sectional	• An adjective attribution task based on 12 stories. Each story was related with one of the following 4 themes: ◦Athletic competence ◦Academic competence ◦Artistic competence ◦Social competence	Overall, overweight characters were less likely to be associated with high athletic ability than average-weight characters. Older participants and boys did it more than younger participants and girls. Overall, overweight characters were less associated with possessing high academic ability. This result was found between 5- to 6-year-olds and 7- to 8-year-olds but not in 9- to 10-year-olds. No gender differences were noted. Overall, good artistic ability was less attributed to overweight figures. However, among 9- to 10-year-olds there was no association between weight and artistic ability. No gender differences were noted. Children 5–8 years old were significantly less likely to associate high social ability with overweight children. However, children 9–10 years old tended to associate high social abilities with overweight children and low social abilities with average-weight children. No gender differences were noted.	• The sample size • The lack of BMIs • The lack of children's perceived body size • The lack of implicit measures
Holub, 2008	USA	To investigate: • The relationship between body size perception and children's anti-fat attitudes	69 children (61% girls and 39% boys) between 3 and 6 years old. Concerning BMI, BMI z-score ranged from -3.02 to 3.27. Regarding ethnicity, there were 97% Caucasian, 1.5% African American and 1.5% Asian. Children' socio-economic background was between $55,000 and $75,000.	Cross-sectional	• BMI • The figure rating scale (with figures A, D, and G only) (Collin's, 1991) to assess children's personal body size perception. • An adjective attribution task: children were shown three figures (A, D, G) of the rating scale (Collin's, 1991) and were asked to attribute to them 5 pairs of adjectives (nice/mean, smart/stupid, has friend/has no friend, neat/sloppy, cute/ugly).	Neither perceived nor body size ratings were related to children's attitudes toward the thinnest figure. Young children who perceived themselves as heavy had less negative attitudes toward overweight children. Self-reported body size correlates more than BMI with anti-fat attitudes.	• The sample was too homogeneous in terms of ethnicity and socio-economic status • The use of Collins' figure rating scale. Collins' figures do not correspond to specific BMI The use of Collins' figure rating scale with children younger than 7 years old. There is no data available for this age • The study design. A longitudinal study would be better • Considering participants as a large group instead of dividing them into different categories
Hansson et al., 2009	Sweden	To investigate: • Anti-fat stereotypes and prejudice in relation to children's sex, sex of the silhouette figure, socio-economic status, place of residence (urban vs. rural) and BMI.	1,409 (647 boys and 762 girls) all 10 years old. Concerning BMI, 141 children were underweight, 1,128 normal weight and 140 overweight and obese. Regarding ethnicity, 95% of children were Caucasian, 5 were Asian, African or South American. Concerning socio-economic status, 456 participants had a high socio-economic background while 953 had a low one.	Cross-sectional	• BMI • An adjective attribution task. Children were asked to rate 6 silhouettes on 19 adjectives (kind, joyful, good, fast, healthy, satisfied, hard-working, honest, happy, loved, clean, popular, good-looking, sloppy, lazy, coward, lonely, slow, strange, stupid, different). The silhouettes used were figures A, D, and I of the Collins' scale (Collins, 1991) both for boys and girls.	The prejudice against obesity was present and not influenced by children's own body size. Any greater degree of obesity prejudice against girls than against boy was discovered. However, boys were more positive to the thin girl figure than the girls themselves. Moreover, children with high socioeconomic status were more likely to show obesity prejudice than children with low socioeconomic status.	• The use of Collins' figure rating scale. Collins' figures do not correspond to specific BMI
Koroni et al., 2009	Greece	To investigate: • Elementary school students' attitudes toward obesity in Greece	1,861 Greek children (934 girls and 927 boys) aged between 10 and 11 years. Regarding BMI, there were 14.1% underweight, 17.7% normal weight, 21.6% overweight and 25.5% obese children.	Cross-sectional	• BMI • The rank-order preference task ideated by Richardson and Royce, 1968. Children were shown 6 figures representing a thin healthy child, an overweight child and 4 children with different forms of disabilities or disfigurements (a child on crutches, a child in a wheelchair, a child with no left hand and a child with a large scar on the left side of the face.) Then they were asked to rank them from the first to the least preferred.	Elementary school students in Greece showed strong anti-fat attitudes against obese peers. All children, regardless of their own body weight, tend to hold negative attitudes toward obese children. Gender differences were discovered in five out of six rankings. Greek boys disliked more functional disabilities (“wheelchair” and “hand”), while Greek girls disliked more appearance problems (“face” and “obese”).	• The ethnic homogeneity • Lack of body children's perceived body size
Askevis-Leherpeux and Schiaratura, 2009	France	To investigate: • If females and males show different grades of anti-fat attitudes toward peers. • If overweight children are considered responsible for their weight and can change their body size	65 (37 boys and 38 girls) between 5 and 6 years old. Concerning body size, 2 girls were considered as overweight by the researchers Regarding ethnicity, there were 45% European and 40% Maghrebi children. Concerning socioeconomic status, all participants had a low socioeconomic background.	Cross-sectional	• Rank order preference task: children were shown 3 series of 6 pictures each representing a specific child (boy or girl). The 3 series diverged because each specific child was once thin, once average and once overweight. • A questionnaire to assess children's opinions about responsibility and controllability of weight.	Both males and females firstly preferred the average peer, secondly the thin one and thirdly the obese peer. However, girls refused obese peers more than boys did. Both boys and girls believed that overweight peers are fat because they eat too much. However, regarding the solutions, girls underline more than boys that children can modify their weight doing more exercise and eating less.	• The sample size • The lack of BMIs • The use of figures that don't correspond to specific BMI
Solbes and Enesco, 2010	Spain	To explore: • The presence of explicit and implicit anti-fat attitudes and their links with participants' personal body attitudes • If explicit and implicit anti-fat attitudes predict children's playmate preference	120 children (60 boys and 60 girls) between 6 and 11 years old, divided into three different age groups: • 40 children from first grade (6–7 years old, mean age = 6.9) • 40 from third grade (8–9 years old, mean age = 8.9) • 40 from fifth grade (10–11 years old, mean age = 10.8) Regarding ethnicity, all participants were white Spanish. Concerning socio-economic status, all children had an upper-middle socio-economic background.	Cross-sectional	3 measure to assess explicit attitudes toward weight: • Simple preferences and rejections task: children were asked to indicate their preferences and rejections toward 4 photos of boys (2 average and 2 overweight) and 4 girls (2 average and 2 overweight) • Sociometric task: children were asked to choose and reject possible partners to carry out different activities (working together, playing and going to a birthday party) • Adjective attribution task similar to the storyline method developed by Cramer and Steinwert (1998) 1 measure to assess personal body attitudes: • Figure rating scale (Collin's, 1991) 1 measure to assess implicit attitudes toward weight: • Implicit Association Task in a child-oriented version adapted from Baron and Banaji (2006).	All age participants showed important prejudice and stereotypes against overweight children, both at the explicit and implicit levels. However, as they grew older, they reduced their levels of explicit prejudice, but not the intensity of implicit bias.	• The ethnic and socio-economic characteristics of the sample. It was too homogeneous under this point of view • The lack of participants' BMI data
Durante et al., 2014	Italy	To explore: • Children's body size attitudes in relation to the stereotype contents	158 children (79 males and 79 females) of which: • 50 participants (24 males and 26 females) between 6 and 7 years old • 55 participants (30 males and 25 females) between 8 and 9 years old • 53 participants (25 males and 28 females) between 10 and 11 years old.	Cross-sectional	• A rating preference task: children were shown 6 pictures (3 representing a thin, an average and a fat boy and 3 representing a thin, an average and a fat girl) and then were asked some questions created in order to assess: 1) the attitude toward body size; 2) the stereotype content, in terms of competence and warmth • The Crandall Social Desirability Test for Children (CDSDTC short form, 1965) to assess social desirability in the fifth-grade children.	Children showed the most positive attitude toward average-weight targets and the most negative attitude toward overweight targets. Both the preference for average weight and dislike for the overweight children lowered from the first to the fifth grade children due to the increase of social desirability concerns. Regarding gender, the authors found that children favored their own gender even if average-weight and overweight female targets were evaluated less favorably than their male counterparts. Concerning the stereotypes' content, overweight targets were perceived as the least competent but warmer than the thin targets. Thin figures were judged as more competent than warm, while average-weight targets were perceived as both competent and warm. Anti-fat attitudes declined based on participants' age and level of social desirability.	• The CDSDTC was administered only to fifth-grade children • Thin and average figures were more similar to the average than the heavy one. • The lack of BMIs
Kornilaki, 2014	Greece	To investigate: • The content and the extent of obesity bias in preschool children • The effects of body size on children's anti-fat bias	85 nursery children (42 boys and 43 girls) between 58 and 68 months of age. Concerning BMI, there were 56.5% normal weight and 43.5% obese children. Regarding ethnicity, participants were predominantly Greek.	Cross-sectional	BMI and 2 measures to assess the obesity bias: • An adjective attribution task based on the storyline method developed by Cramer and Steinwert (1998). Children were asked to attribute social, school, athletic, art and self-competence to a story's characters different in body size.• The playmate preference task realized through the use of three figure (A, D, G) of the Collins' figure rating scale.	Preschool children tended to assign negative qualities to obese figures and positive characteristics firstly to normal weight and secondly to thin figure silhouettes. Similarly, in the playmate preference task, the average and the thin figures were the most wanted, both by normal and obese children. Therefore, obesity bias resulted present at preschool age and independent of children's body size.	• The use of Collins' figure rating scale. Collins' figures do not correspond to specific BMI • The use of Collins' figure rating scale with children younger than 7 years old. There is no data available for this age
Kornilaki, 2015	Greece	To investigate: • How children of various weight perceive their body size • If children's BMI or perceived body size is connected with their anti-fat views • How overweight and obese children perceive themselves and if their body perception determines their anti-fat attitudes	414 children of which: • 131 were 5–6 years old (68 boys and 63 girls) • 152 were 7–8 years old (76 boys and 76 girls) • 131 were 9–10 years old (56 girls and 75 boys Concerning BMI, there were 3.6% underweight, 60% normal weight, 23.9% overweight, and 12.3% obese children. Regarding the ethnicity, all participants were white Caucasian. Children's socio-economic background varied.	Cross-sectional	• BMI • The figure rating scale (with 7 figures) (Collins, 1991) to assess children's perceived body size. An adjective attribution task, based on the storyline method developed Cramer and Steinwert, 1998 to assess obesity bias.	Children tended to identify themselves with the low average figures of Collins' (1991) scale, irrespective of their own body size. Girls chose the lighter figures significantly more often than boys. Weight status affected the accuracy of body size perception. Average weight children were the most accurate while the overweight and obese children underestimated their weight and the underweight overestimated it. Obesity bias is strengthened with age and it is influenced by perceived rather than by actual body size. Only the overweight and obese children that identified themselves as heavy had lower obesity bias scores, but still had them.	• The use of Collins' figure rating scale. Collins' figures do not correspond to specific BMI • The use of Collins' figure rating scale with children younger than 7 years old. There is no data available for this age
Harriger, 2015	USA	To explore: • The age differences in the development of body-size stereotyping	102 girls between 3 and 5 years old of which 35 were 3 years old, 37 were 4 years old and 30 were 5 years old. Regarding ethnicity, there were 62% white, 24% Hispanic and 14% of children of other minorities.	Cross-sectional	2 measures to assess the anti-fat bias • An adjective attribution task: children were shown 3 figures (A, D, G of the figure rating scale by Collins, 1991) then asked to evaluate them on 6 pairs of adjectives (nice/mean, smart/stupid, neat/sloppy, has friend/has no friend, cute/ugly, quiet/loud) • A friend selection task realized through the use of the same figures utilized in the first task.	Preschool-aged girls did engage in body-size stereotyping. They attributed more negative adjectives to fat targets and more positive adjectives to thin targets. They were also more likely to select a thin figure as their playmate or their best friend than a fat figure. In a number of tasks no age differences existed, but follow-up analysis evidenced that 3-year-olds tended to select a fat friend more often compared to 5-year-olds. On the contrary, in the adjective attribution task, younger girls were harsher with fat figures, compared to the older ones.	• The lack of boys • The lack of BMIs • The lack of children's perceived body size
Burmeister et al., 2016	USA	To explore: • Children's reactions to a set of images representing active or inactive obese children • Gender differences in that task	44 (45.5% girls and 54.5% boys) whose mean age was 4.7 (*SD* = 0.49). Concerning BMI, the mean percentile ranking was 73.30 (*SD* = 25.93). Regarding ethnicity, there were 89.5% white, 5.3% black and 5.3% multiracial children.	Cross-sectional	• BMI • A rating preference task: children were shown 6 images (3 representing active obese peers and 3 representing inactive obese peers). Then participants were asked to answer a series of questions created to assess their ratings through the use of a 4-point scale.	Boys and girls rated children with obesity in images differently depending on how they were depicted. Boys had more favorable impressions of overweight children shown in active roles while girls had negative impressions of overweight children portrayed both in active and passive roles.	• The size sample • The ethnic aspect of the sample.
Ruffman et al., 2016	New Zealand	To explore: • If infants and toddlers exhibit anti-fat bias	70 mother-child dyads comprising 18 young infants (*M* = 6.99 months), 19 older infants (*M* = 11.11 months), 16 young toddlers (*M* = 28.81 months) and 17 older toddlers (*M* = 32.29 months).	Cross-sectional	• Children's looking biases to assess the babies looking preference. Infants and toddlers were shown 10 pairs of figures containing an average and an obese figure. • The Crandall's Anti-fat Attitudes Questionnaire (1994) to assess mothers' explicit anti- prejudice. • Maternal and paternal BMI. • Children's television viewing.	Older toddlers' preferential looking was significantly influenced by maternal attitudes, so that the children of mothers with anti-fat attitudes could express clear preferences for normal weight figures, while infants (*M* = 11 months) had a bias for looking at the obese figures.	• The sample size • The lack of paternal anti-fat bias • The ethnic homogeneity of the sample

The papers identified produced a sample size ranging from very small (*n* = 24 children, Counts et al., 1986) to large-scale studies (*n* = 1, 861 children, Koroni et al., 2009). The age range covered 6 months—11 years old within the articles and all but 1 study included both boys and girls. The exception was Harriger (2015) an girl-only sample.

From an ethnical point of view, the majority of the studies included above all white participants. However, other ethnicities are represented as part of the sample in 6 papers (Goldfield and Chrisler, 1995; Kraig and Keel, 2001; Holub, 2008; Askevis-Leherpeux and Schiaratura, 2009; Hansson, 2009; Harriger, 2015; Burmeister et al., 2016). All studies are cross-sectional.

### Anti-fat stigma and body size

The search revealed 8 papers addressing the relationship between anti-fat stigma and body size (Counts et al., 1986; Cramer and Steinwert, 1998; Kraig and Keel, 2001; Holub, 2008; Hansson et al., 2009; Koroni et al., 2009; Kornilaki, 2014, 2015; Table 1).

#### Instruments used

Regarding the measurement of anti-fat stigmatization, 7 out of 8 studies used an adjective attribution task (Counts et al., 1986; Cramer and Steinwert, 1998; Kraig and Keel, 2001; Holub, 2008; Hansson et al., 2009; Kornilaki, 2014, 2015), 2 a playmate preference task (Cramer and Steinwert, 1998; Kornilaki, 2014) 1 a rank order preference task (Koroni et al., 2009) and 1 a personal body attitude task (Cramer and Steinwert, 1998)^2^.

Concerning the measurement of body size, 6 studies calculated participants' BMI while one (Counts et al., 1986) evaluated children's body size by eye. Additionally, 2 also investigated children's perceived body size (Holub, 2008; Kornilaki, 2015).

#### Findings

Despite the sample and the methodological differences, all studies concluded that actual body size—precisely measured in terms of BMI or even calculated visually by researchers—does not affect children's anti-fat attitudes significantly. Thus, researchers who try to correlate body size and anti-fat bias discovered that overweight and obese children stigmatized their in-group peers as well as thin and average children did or even more so (Cramer and Steinwert, 1995).

However, this unexpected result appears mitigated by the conclusions reached by the two studies that added the perceived body size information to the BMI data. Interestingly, both Holub (2008) and Kornilaki (2015) found a low correlation between children's self-perceived and actual body size especially in overweight and obese children. Specifically, they discovered that the latter categories of children tended to underestimate their body size.

In particular, Kornilaki (2015) observed that the great majority of fat children considered themselves as normal (77.8%) or even thin (9.1%), with only 13.1% correctly identifying with the heaviest silhouette. Moreover, she ascertained that overweight accuracy considerably improved with age (from 11.8% at the age of 5–6 to 24.2% at the age of 9–10) but it steadily remained lower than the one reached by average peers, which went from 68.2% at the age of 5–6 to 86.8% at the age of 9–10. Thus, at every age, more than half of the overweight children identified themselves as average and stigmatized the fat silhouette as if it represented an out-group member. Only the minority of children who recognized their actual body size showed less anti-fat stigma, but still presented it.

Similar conclusions were found by Holub (2008) and stated that perceived body size is the only univariate predictor of anti-fat attitudes in overweight children.

### Anti-fat stigma and sex

The search revealed 13 papers addressing the relationship between anti-fat stigma and sex (Lerner and Gellert, 1969; Counts et al., 1986; Goldfield and Chrisler, 1995; Cramer and Steinwert, 1998; Turnbull et al., 2000; Kraig and Keel, 2001; Penny and Haddock, 2007a,b; Askevis-Leherpeux and Schiaratura, 2009; Hansson, 2009; Koroni et al., 2009; Durante et al., 2014; Burmeister et al., 2016; Table 1).

#### Instruments used

Relative to the measurement of anti-fat stigmatization 6 studies utilized an adjective attribution task (Counts et al., 1986; Cramer and Steinwert, 1998; Turnbull et al., 2000; Kraig and Keel, 2001; Penny and Haddock, 2007b; Hansson, 2009), 3 a friend selection task (Goldfield and Chrisler, 1995; Cramer and Steinwert, 1998; Penny and Haddock, 2007a) 2 a rank order task (Askevis-Leherpeux and Schiaratura, 2009; Koroni et al., 2009) 2 a rating preference task (Durante et al., 2014; Burmeister et al., 2016), and 3 a body size preference task (Lerner and Gellert, 1969; Goldfield and Chrisler, 1995; Cramer and Steinwert, 1998).

Regarding the measurement of body size, 3 studies calculated participants' BMI (Hansson, 2009; Koroni et al., 2009; Burmeister et al., 2016) while 2 (Counts et al., 1986; Askevis-Leherpeux and Schiaratura, 2009) evaluated children's body size visually.

Moreover, Askevis-Leherpeux and Schiaratura (2009) administered a questionnaire to assess children's opinions about responsibility and controllability of weight and Durante et al. (2014) administered The Crandall Social Desirability Test for Children (1965).

#### Findings

Firstly, all studies, regardless of the sample and the methodological differences, evidenced that both male and female children as young as 2 years old exhibited anti-fat attitudes toward their overweight and obese peers. Thus, the overweight or obese silhouettes/drawings/photos were overall associated with negative adjectives and less preferred as friends than the normal weight ones.

However, 5 studies found no significant differences between sexes in the degree of anti-fat attitudes shown actively by boys and girls, in the degree of anti-fat bias directed toward male and female stimuli and even in the attitudes expressed toward average and thin figures. Conversely, 8 studies identified sex differences.

In particular, 2 studies (Kraig and Keel, 2001; Hansson et al., 2009) revealed that there was a sex difference regarding the socially valued body size. In particular, Kraig and Keel (2001) noticed that girls didn't seem to be the object of a greater degree of anti-fat bias but, interestingly, when evaluating boys, children distinguished between overweight boys—who were devalued—and normal and thin males—who were both valued. Differently, when evaluating girls, children distinguished between thin peers—who were the most valued—and average and overweight females—who were more devalued.

Hansson et al. (2009), instead, found that boys rated thin girls more positively than the girls themselves did. Thus, both studies underlined the fact that boys could in some sense press girls to value being thin.

Moreover, 6 studies identified a gender difference in anti-fat bias. Turnbull et al. (2000) as well as Penny and Haddock (2007a) found that fat stigma was stronger toward female targets rather than male ones. Moreover, the second group of authors discovered another interesting trend in children, asking them to evaluate a male or female figure who was surrounded by average or overweight characters. In fact, in theory, children should not take into consideration the background characters, but in practice it emerged that the target and background size interacted and influenced judgments regarding female targets. More precisely, an average-weight female target was evaluated more negatively on an overweight background and an overweight female target was evaluated more negatively on an average-weight background.

Other authors revealed that girls exhibited higher more deeply-rooted forms of anti-fat bias than boys. Askevis-Leherpeux and Schiaratura's study (2009) showed that, although boys and girls believed that overweight peers were fat because they ate too much, girls rejected chubby peers more than boys. Moreover, females thought that losing weight depended on willpower and recommended more physical exercises to girls and dieting to boys.

Burmeister et al. (2016) demonstrated that males and females rate obese peers in various images differently depending on how they are portrayed. Specifically, while boys value more positively obese children depicted while doing dynamic activities than doing passive ones, girls are not influenced by them. This phenomenon may be explained considering that boys might have more favorable attitudes toward active overweight children because for males the thin-ideal is mixed with the muscular one. For girls, instead, independently from how they are depicted, obese children still remain obese and for this reason, deplorable.

Similar conclusions were reached by Koroni et al. (2009). These authors, asking Greek children to rank 6 figures representing a thin healthy child, an overweight child and 4 children with different forms of disabilities or disfigurements (a child on crutches, a child in a wheelchair, a child with no left hand and a child with a large scar on the left side of the face), evidenced that boys disliked more functional disabilities (“wheelchair” and “hand”), while girls disliked more appearance problems (“face” and “obese”).

Finally, Durante et al. (2014) found that usually children favored their own gender even if average-weight and overweight female targets were evaluated less favorably than their male counterparts. Thus, in-group favoritism was less evident in girls when rating both average and chubby peers.

### Anti-fat stigma and age

Generally, all research papers may be used to analyze the relationship between anti-fat attitudes and age. However, from a more analytic point of view, 8 articles address this issue more precisely (Cramer and Steinwert, 1998; Turnbull et al., 2000; Penny and Haddock, 2007b; Solbes and Enesco, 2010; Harriger, 2015; Kornilaki, 2015; Durante, 2016; Ruffman, 2016; Table 1).

#### Instruments used

Concerning the measurement of anti-fat stigmatization, 6 studies utilized an adjective attribution task (Cramer and Steinwert, 1998; Turnbull et al., 2000; Penny and Haddock, 2007b; Solbes and Enesco, 2010; Harriger, 2015; Kornilaki, 2015), 2 a playmate selection task (Cramer and Steinwert, 1998; Harriger, 2015), 2 a personal body size attitude task (Cramer and Steinwert, 1998; Solbes and Enesco, 2010), 1 a simple preferences and rejections task, a socio-metric task and the Implicit Association Task (Solbes and Enesco, 2010),1 a rating preference task and 1 a looking bias task (Ruffman, 2016).

Regarding the measurement of body size, only 1 study calculated participants' BMI (Kornilaki, 2015).

Moreover, Durante et al. (2014) administered The Crandall Social Desirability Test for Children (1965).

#### Findings

First, all studies evidenced the presence of anti-fat stigma, demonstrating that it is very pervasive and deeply rooted.

However, analyzing this phenomenon over time the following trend emerges:

In the first place, according to Turnbull et al. (2000) and Ruffman et al. (2016) the anti-fat attitude emerges during the second year of life. In fact, both the adjective attribution task used by the first group of authors and a looking preference task used by the second group of authors revealed this.

Regarding the age group between 3 and 5 year olds, Cramer and Steinwert (1998) recorded that both in the adjective attribution and in the playmate preference tasks fat stigmatization was stronger in older children. Contrarily, Harriger (2015), with an only-girl sample, discovered that in the adjective attribution task younger girls were harsher on fat figures compared to the older ones. However, in the friend selection task, 3-year-old girls selected fat friends more frequently than 5-year-old ones.

Concerning the age group between 5 and 10, Penny and Haddock (2007b) and Kornilaki (2015) reached opposite conclusions. Penny and Haddock found that children 5–8 were less likely to associate being overweight with athletic, artistic and social abilities. On the contrary, this phenomenon didn't emerge in children 9–10, who even tended to attribute social abilities to overweight peers. Conversely, Kornilaki revealed that obesity stigma increased with age.

Finally, concerning children between 6 and 11 both Solbes and Enesco (2010) and Durante et al. (2014) found that anti-fat stigma reduced with age. However, Durante et al. underlined that this phenomenon was directly proportional not only to age but also to the increase of social desirability. Similarly, Solbes and Enesco revealed that as children grew older, they reduced their level of explicit prejudice but not the intensity of the implicit bias.

## Conclusion

Regarding the first objective of this paper, it emerged that overweight and obese children show in-group favoritism and thus less anti-fat attitudes only when they correctly perceive their actual body size (Holub, 2008; Kornilaki, 2015). However, according to Kornilaki the majority of fat children don't accurately identify their body even at 9–10 years old. Therefore, children's in-group favoritism may be a rare phenomenon.

Concerning the second aim, the question remained substantially unanswered because 5 articles posit no gender differences (Lerner and Gellert, 1969; Counts et al., 1986; Goldfield and Chrisler, 1995; Cramer and Steinwert, 1998; Penny and Haddock, 2007a) while 8 noticed that girls stigmatize more than boys and are even more often the object of fat prejudice compared to their male peers (Kraig and Keel, 2001; Askevis-Leherpeux and Schiaratura, 2009; Hansson, 2009; Koroni et al., 2009; Durante et al., 2014; Burmeister et al., 2016) However, overall, it can be noted that 4 out of the 5 studies that didn't perceive sex differences were published before 2000.

Finally, concerning the third purpose, most of the studies considered partially or fully corroborated Aboud's Cognitive-Developmental Theory (1988, 2003). In fact, in the adjective attribution task, both Cramer and Steinwert (1998) and Kornilaki (2015) noticed an intensification of the anti-fat bias between 3 and 5 years old, while in the playmate selection task only Cramer and Steinwert recognized it. Moreover, except for Kornilaki (2015), the other studies examined (Penny and Haddock, 2007b; Solbes and Enesco, 2010; Durante et al., 2014) observed an explicit decrease in anti-fat bias starting at 9 years old. However, as noticed by Penny and Haddock, this change may be ascribed to the increase of social desirability and as noticed by Solbes and Enesco, it could be only exterior but not interior.

Three among the possible future developments of this mini-review, which demonstrates the limitation of using a small number of articles written in different time periods, could be: (1) expand the research; (2) consider the effect of the research contexts on the results; (3) more deeply analyze the methodologies used and their impact on the results.

## Author contributions

RD handled the global structure of the work and the literature selection. LC collaborated in the process of literature selection.

### Conflict of interest statement

The authors declare that the research was conducted in the absence of any commercial or financial relationships that could be construed as a potential conflict of interest.

## Footnotes

^1^The decision to place the cut off at 11 years old is dictated by the desire to analyze the phenomenon before adolescence.

^2^It's important to notice that, although the methodologies used are often nominally similar, in practice they are very heterogeneous in terms of the number of adjectives chosen, semantic field they belong to and stimulus figures utilized.
